# Interleukin-1 receptor accessory protein blockade limits the development of atherosclerosis and reduces plaque inflammation

**DOI:** 10.1093/cvr/cvae046

**Published:** 2024-04-02

**Authors:** Megan Mulholland, Marie A C Depuydt, Gabriel Jakobsson, Irena Ljungcrantz, Andrietta Grentzmann, Fong To, Eva Bengtsson, Elin Jaensson Gyllenbäck, Caitríona Grönberg, Sara Rattik, David Liberg, Alexandru Schiopu, Harry Björkbacka, Johan Kuiper, Ilze Bot, Bram Slütter, Daniel Engelbertsen

**Affiliations:** Department of Clinical Sciences, Cardiovascular Research—Immune Regulation, Lund University, Malmö, Sweden; Leiden Academic Centre for Drug Research, Division of Biotherapeutics, Leiden University, Leiden, The Netherlands; Department of Translational Medicine, Cardiac Inflammation, Lund University, Malmö, Sweden; Department of Clinical Sciences, Cardiovascular Research—Immune Regulation, Lund University, Malmö, Sweden; Department of Clinical Sciences, Cardiovascular Research—Immune Regulation, Lund University, Malmö, Sweden; Department of Clinical Sciences, Cardiovascular Research—Matrix and Inflammation in Atherosclerosis, Lund University, Malmö, Sweden; Department of Clinical Sciences, Cardiovascular Research—Matrix and Inflammation in Atherosclerosis, Lund University, Malmö, Sweden; Department of Biomedical Science, Malmö University, Malmö, Sweden; Biofilms—Research Center for Biointerfaces, Malmö University, Malmö, Sweden; Cantargia AB, Lund, Sweden; Cantargia AB, Lund, Sweden; Department of Clinical Sciences, Cardiovascular Research—Immune Regulation, Lund University, Malmö, Sweden; Cantargia AB, Lund, Sweden; Cantargia AB, Lund, Sweden; Department of Translational Medicine, Cardiac Inflammation, Lund University, Malmö, Sweden; Department of Clinical Sciences, Cardiovascular Research—Cellular Metabolism and Inflammation, Lund University, Malmö, Sweden; Leiden Academic Centre for Drug Research, Division of Biotherapeutics, Leiden University, Leiden, The Netherlands; Leiden Academic Centre for Drug Research, Division of Biotherapeutics, Leiden University, Leiden, The Netherlands; Leiden Academic Centre for Drug Research, Division of Biotherapeutics, Leiden University, Leiden, The Netherlands; Department of Clinical Sciences, Cardiovascular Research—Immune Regulation, Lund University, Malmö, Sweden

**Keywords:** Atherosclerosis, Immunomodulation, IL-1, Inflammation, IL1RAP

## Abstract

**Aims:**

The interleukin-1 receptor accessory protein (IL1RAP) is a co-receptor required for signalling through the IL-1, IL-33, and IL-36 receptors. Using a novel anti-IL1RAP-blocking antibody, we investigated the role of IL1RAP in atherosclerosis.

**Methods and results:**

Single-cell RNA sequencing data from human atherosclerotic plaques revealed the expression of IL1RAP and several IL1RAP-related cytokines and receptors, including *IL1B* and *IL33*. Histological analysis showed the presence of IL1RAP in both the plaque and adventitia, and flow cytometry of murine atherosclerotic aortas revealed IL1RAP expression on plaque leucocytes, including neutrophils and macrophages. High-cholesterol diet fed apolipoprotein E–deficient (*Apoe^−/−^*) mice were treated with a novel non-depleting IL1RAP-blocking antibody or isotype control for the last 6 weeks of diet. IL1RAP blockade in mice resulted in a 20% reduction in subvalvular plaque size and limited the accumulation of neutrophils and monocytes/macrophages in plaques and of T cells in adventitia, compared with control mice. Indicative of reduced plaque inflammation, the expression of several genes related to leucocyte recruitment, including *Cxcl1* and *Cxcl2*, was reduced in brachiocephalic arteries of anti-IL1RAP-treated mice, and the expression of these chemokines in human plaques was mainly restricted to CD68^+^ myeloid cells. Furthermore, *in vitro* studies demonstrated that IL-1, IL-33, and IL-36 induced CXCL1 release from both macrophages and fibroblasts, which could be mitigated by IL1RAP blockade.

**Conclusion:**

Limiting IL1RAP-dependent cytokine signalling pathways in atherosclerotic mice reduces plaque burden and plaque inflammation, potentially by limiting plaque chemokine production.

## Graphical Abstract

**Graphical Abstract:**
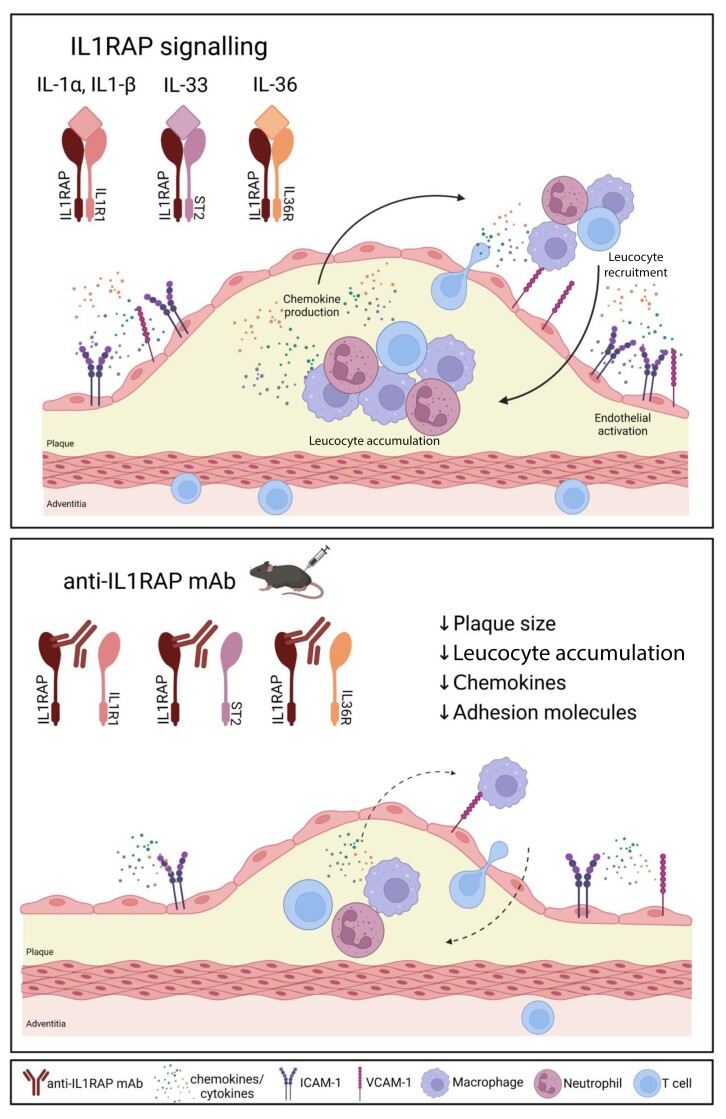

**Time of primary review: 54 days**

## 1. Introduction

The vulnerable atherosclerotic plaque is characterized by chronic non-resolving inflammation.^1^ Several studies have evaluated the effect of anti-inflammatory immunomodulatory therapy on cardiovascular disease burden.^2,3^ The CANTOS trial demonstrated that neutralization of interleukin-1β (IL-1β) using canakinumab results in reduced incidence of cardiovascular events compared with placebo.^4^ While providing proof-of-principle of the biological importance of the IL-1 pathway in the development of atherosclerosis, the reduction in cardiovascular events did not translate to a net benefit in survival, and the development of canakinumab for treatment of cardiovascular disease was not continued. Thus, more effective strategies to interfere with cytokine-induced plaque inflammation are needed.

IL-1 receptor accessory protein (IL1RAP, also called IL-1R3 or IL-1RAcP) is a co-receptor required for signalling of several IL-1 family cytokines (IL-1α, IL-1β, IL-33, IL-36α, IL-36β, and IL-36γ). In atherosclerosis, the role of IL-1β has been thoroughly studied,^5,6^ but there is evidence that other IL1RAP-related cytokines are implicated in disease progression. Several studies have identified IL-1α as a promoter of plaque inflammation and atherosclerosis,^7,8^ and investigations into the role of IL-33 in atherosclerosis have yielded divergent findings.^9,10^ IL-36 has been suggested to play a role in the response to myocardial ischaemia,^11^ and reports have suggested a pro-inflammatory effect of IL-36 in atherosclerosis as well.^12,13^

A humanized antibody targeting IL1RAP and blocking IL-1, IL-33, and IL-36 signalling (CAN10, Cantargia AB) is currently under development for treatment of inflammatory and fibrotic diseases, such as myocarditis and systemic sclerosis. Given that several pro-atherogenic IL-1 family cytokines signal through IL1RAP, blockade of this pathway may represent a more comprehensive therapeutic strategy to limit plaque inflammation. To test this hypothesis and investigate the role of IL1RAP in atherosclerosis, we treated apolipoprotein E–deficient (*Apoe^−/−^*) mice with a non-depleting IL1RAP-blocking antibody. Results demonstrate that treatment with an IL1RAP-blocking antibody limits plaque development and plaque inflammation.

## 2. Methods

### 2.1 Human material

Human carotid artery plaques were collected from 18 patients (14 male, 4 female) that underwent carotid endarterectomy surgery as part of Athero-Express, an ongoing biobank at the University Medical Centre Utrecht (UMCU).^14^ The study was approved by the Medical Ethics Committee of the UMCU (study approval number: TME/C-01.18, protocol number 03/114). For flow cytometric analysis, whole blood and carotid atherosclerotic plaques were obtained from four patients that underwent carotid endarterectomy surgery at the Haaglanden Medical Center (HMC), Westeinde, The Hague, The Netherlands (study approval number: Z19.075, protocol number NL71516.058.19, approved by the Medical Ethics Committee of the HMC). All blood samples were collected by venipuncture prior to surgery. Atherosclerotic plaque specimens were obtained from primary endarterectomy surgeries, and restenotic plaques were excluded due to their different plaque compositions as compared with primary atherosclerotic plaques. All studies were performed in accordance with the Declaration of Helsinki. Informed consent was obtained from all subjects involved in the study.

### 2.2 Mice

Wild-type mice (C57Bl/6J) were bred in-house or purchased from Janvier Laboratories, and *Apoe^−/−^* mice (B6.129P2-Apoe^tmlUnc^/J) were bred in-house or purchased from Jackson Laboratory. Mice utilized in the *in vivo* experiment with anti-IL1RAP antibody treatment were all purchased from Jackson Laboratory. Mice were terminated using *i.p.* injection of ketamine/xylazine (150 and 50 mg/kg) followed by exsanguination via cardiac puncture. Animal experiments were approved by the local ethics committee (ethical permits #8997-18) and were in compliance with EU guidelines (directive 2010/63/EU for the protection of laboratory animals).

### 2.3 Single-cell RNA sequencing

Single-cell RNA sequencing on human carotid plaques was performed as previously described.^15^ In brief, human carotid plaques were digested; live cells were sorted and processed using the SORT-seq platform. Data analysis was performed using the Seurat pipeline.

### 2.4 Flow cytometry of human carotid artery plaques and peripheral blood mononuclear cells

Single cells from carotid artery plaques were isolated by enzymatic digestion, and peripheral blood mononuclear cells (PBMCs) were isolated by density centrifugation and frozen in liquid nitrogen until use. Single-cell suspensions from blood and plaque were thawed and washed in RPMI 1640 containing 10% foetal bovine serum. Thawed plaque cells and PBMCs were washed and stained for 30 min at 4°C with extracellular antibodies/dyes (see Supplementary material online, *Methods*, for antibody list) and analysed with FlowJo version 10.7.

### 2.5 IL1RAP antibody

The anti-IL1RAP antibody used is a non-depleting blocking mouse IgG2a anti-IL1RAP antibody (clone: 3A9) with a LALA-PG mutation to reduce Fc receptor binding^16^ (Icosagen, Tartu, Estonia). Optimal dosing of IL1RAP-blocking antibody was determined in pharmacokinetics studies to be 20 mg/kg loading dose and 10 mg/kg twice a week for subsequent doses, when given intraperitoneal (data not shown). Control isotype was anti-HEL mouse IgG2a-LALA-PG (Icosagen, Tartu, Estonia). All anti-IL1RAP reagents and isotype control antibodies were provided by Cantargia AB, Sweden.

### 2.6 *In vivo* experiments

Female *Apoe^−/−^* mice, age 10–11 weeks at the start of experiment, were kept on a high-cholesterol diet (HCD; 0.21% cholesterol, 21% butter fat) for a total of 10 weeks, and biweekly *i.p.* injections of anti-IL1RAP antibodies or isotype control IgG (*n* = 14/group) were administered during the final 6 weeks of HCD. Mice were terminated 2 days after the final dose. To study effects of IL1RAP on cholesterol levels in wild-type mice, male C57Bl/6 mice, age 10–11 weeks at the start of experiment, were kept on normal chow diet. Mice were administered anti-IL1RAP or isotype control IgG antibodies (*n* = 7/group) for a total of 3 doses. Mice were terminated 2 days after the final dose. To determine effects of anti-IL1RAP treatment on dendritic cell function, HCD-fed *Apoe^−/−^* mice were administered biweekly injections of either anti-IL1RAP or isotype control IgG (*n* = 7/group) for 20 days until termination. All mice were randomly assigned into treatment groups and cage-mates were used as controls.

### 2.7 Histology

Murine hearts were frozen and mounted in O.C.T. compound (VWR) for sectioning of aortic roots. Cross-sections were collected starting at the beginning of the root at 6 μm and continued for a total distance of 600 μm. To assess lipid content and plaque area, cross-sections were stained with Oil Red O and counterstained with Harris’ haematoxylin. Plaque collagen content was assessed with Masson’s trichrome staining. Methods of immunohistochemical and immunofluorescence stainings are shown in the Supplementary material online, *Methods*. All histology were quantified using QuPath v0.2.3.^17^

### 2.8 Tissue preparation and flow cytometry

At termination, aorta, blood, plasma, spleen, iliac aortic lymph nodes, brachiocephalic artery (BCA), and hearts were collected. Blood was collected into EDTA-coated syringes (0.5 M EDTA, eBioscience) via cardiac puncture. Red blood cells were removed from blood and spleen samples with Ammonium-Chloride-Potassium (ACK) lysis buffer (ThermoFisher). Aortas were digested by cutting into small pieces within a digestion mix (450 U/mL collagenase I, 125 U/mL collagenase XI, 60 U/mL DNAse I, 60 U/mL hyaluronidase I, 20 mM HEPES buffer) and incubating in this mix for 1 h at 37°C, shaking at 300 RPM. For measurement of T cell cytokine production, single-cell suspensions of splenocytes were incubated in DMEM supplements with 10% foetal bovine serum (FBS, Gibco) and PMA/ionomycin with Brefeldin A (Cell Stimulation Cocktail with Brefeldin A, BioLegend) for 4 h at 37°C. Flow cytometry was performed on a Gallios flow cytometer (Beckman Coulter Inc., GA, USA) and cytometric analysis with FlowJo software v10.8.0 (Tree Star Inc., OR, USA).

### 2.9 RNA extraction and real-time PCR

BCAs were pooled (*n* = 2/pool) and homogenized with a mechanical homogenizer in Trizol (TRI Reagent Solution, Invitrogen). After lysis of cells, chloroform was added to each suspension, shaken by hand, and then centrifuged to achieve phase separation. Aqueous phase was removed and added to 100% isopropanol and LPA followed by another centrifugation to precipitate the RNA pellet. RNA was then washed with 75% ethanol. Supernatant was removed, and pellet was allowed to air dry for about 1 min. RNA pellet was rehydrated with RNAse-free water and converted to cDNA with high-capacity RNA-to-cDNA kit (Applied Bioscience) according to manufacturer’s guidelines using a SimpliAmp Thermocycler. TaqMan probes were from ThermoFisher Scientific, and qPCR was run on QuantStudio 7 Flex using QuantStudio RT-PCR Software v1. Data are presented as relative fold expression (2^−ΔΔCt^ method) of target genes, normalized to 18S.

### 2.10 Bone marrow–derived macrophage and NIH3T3 fibroblast *in vitro* stimulation

Bone marrow was collected from the tibias and femurs of wild-type C57Bl/6 mice by flushing the marrow out of the bone cavities with PBS. Bone marrow–derived macrophages (BMDMs) were obtained by culturing bone marrow cells with 15% L929-conditioned medium (containing M-CSF) for a total of 7 days. BMDMs (3 × 10^5^ cells per well) were pre-incubated with either anti-IL1RAP antibodies (20 μg/mL) or control isotype IgG (20 μg/mL) for 15 min before addition of cytokines: IL-1α (10 ng/mL, RnD Systems), IL-1β (10 ng/mL, RnD Systems), or IL-33 (10 ng/mL, RnD Systems). For IL-36 stimulation, BMDMs were obtained by culturing bone marrow cells with hG-CSF (100 ng/mL, RnD Systems) for 6 days, followed by overnight incubation with DMEM supplemented with 10 ng/mL hM-CSF in a 96-well plate (4 × 10^4^ cells per well). BMDMs were pre-incubated for 30 min with either anti-IL1RAP antibodies (20μg/mL) or control isotype IgG (20 μg/mL) and hM-CSF (10 ng/mL), TGF-β (10 ng/mL, RnD Systems), and GM-CSF (10 ng/mL, Thermo Fisher)^18^ before addition of a combination of IL-36α, IL-36β, and IL-36γ (200 ng/mL each, RnD Systems; IL-36 stimulation experiments was performed at Redoxis, Lund, Sweden). Cells were incubated at 37°C for 24 h before supernatants were collected and analysed for chemokine release by multiplex immunoassay (Luminex and Eve Technologies).

Murine fibroblast cell line NIH3T3 (ATCC) was utilized to study fibroblast response to IL1RAP family cytokines (experimental work was performed at Truly Labs, Lund, Sweden). NIH3T3 cells (4 × 10^4^ cells per well) were pre-incubated in a 96-well plate with either anti-IL1RAP antibody at 20 (IL-1β, IL-33, IL-36) or 60 ug/mL (IL-1α) or control isotype IgG 20 (IL-1β, IL-33, IL-36) or 60 ug/mL (IL-1α) for 60 min before addition of cytokines: IL-1α (5 pg/mL, RnD Systems), IL-1β (30 pg/mL, RnD Systems), IL-33 (15 ng/mL, PeproTech) IL-36α (15 ng/mL, RnD Systems), IL-36β (8 ng/mL, RnD Systems), or IL-36γ (15 ng/mL, RnD Systems). Cells were incubated at 37°C for 18 h before supernatants were collected and analysed for chemokine release by ELISA (ThermoFischer Scientific).

### 2.11 Plasma lipid, cytokine, and antibody analyses

Total plasma cholesterol was quantified using Infinity^TM^ Cholesterol kit (Thermo Scientific), according to manufacturer guidelines. Plasma cytokines were quantified using a multiplex immunoassay (Milliplex Map Kit, MCYTOMAG-70 K, Millipore Sigma), according to manufacturer guidelines. IgM antibody levels against phosphoryl-choline bovine serum albumin (PC-BSA) and copper-oxidized low-density lipoprotein (Cu-oxLDL) was measured by a custom-made ELISA. In brief, IgM antibody levels against PC-BSA (BioSearch, PC-1011-10) and Cu-oxidized human LDL (Cu-oxLDL) was measured using a custom-made ELISA using a biotinylated detection antibody against mouse IgM (goat anti-mouse IgM, Jackson 115-065-075).

### 2.12 Statistics

Data were tested for normal distribution (Kolmogorov–Smirnov normality test) and analysed with unpaired two-tailed Student’s *t*-test or Mann–Whitney *U* test accordingly, and bars denote mean or median, respectively. Statistical analysis was performed using GraphPad Prism v9.2.0 software (GraphPad Software, CA, USA). A *P* value of <0.05 was considered significant, and *P* values <0.10 are reported.

## 3. Results

### 3.1 RNA expression patterns of IL1RAP-related receptors and cytokines in human atherosclerotic plaque cells

**Figure 1:**
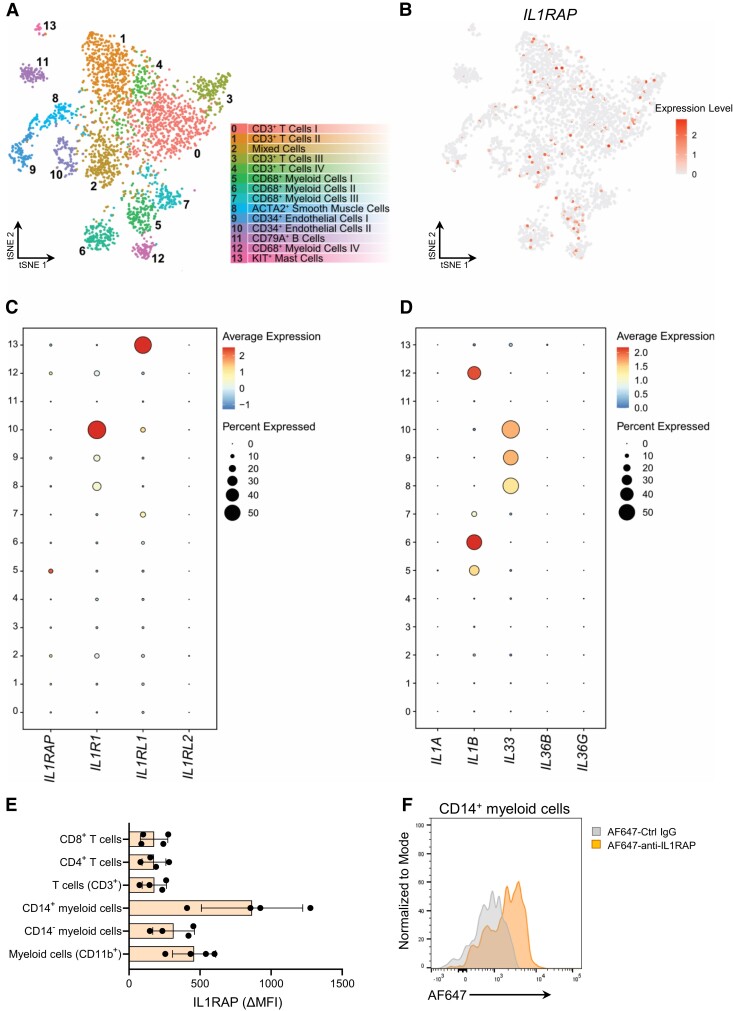
Expression of IL1RAP family receptors and cytokines in human atherosclerotic plaques. Single-cell RNA sequencing data of human carotid plaques (*n* = 18) from patients that underwent carotid endarterectomy. (*A*) tSNE depicting 14 distinct cell clusters consisting of T cells, myeloid cells, endothelial cells, smooth muscle cells, B cells, and mast cells (image from Depuydt *et al*.^15^). (*B*) Feature plot of *IL1RAP* gene expression. Dot plots of gene expression of (*C*) IL1RAP-related receptors and (*D*) IL1RAP-related cytokines. (*E*) Human carotid plaques (*n* = 4) were digested, stained with anti-IL1RAP-Alexa Fluor 647 or isotype control IgG, and analysed for IL1RAP MFI by flow cytometry. ΔMFI defined as the difference in MFI in each cell population between anti-IL1RAP and isotype antibody staining. (*F*) Representative histogram of IL1RAP expression on carotid plaque CD14^+^ myeloid cells.

IL1RAP is a co-receptor necessary for IL-1α, IL-1β, IL-33, and IL-36α/β/γ signalling. To interrogate expression of IL1RAP-associated cytokines and co-receptors on plaque resident cells, we analysed data from single-cell RNA sequencing of human carotid plaque cells. Cellular clusters were defined by unsupervised clustering and assigned a cell type based on transcriptional profile (*Figure 1A*) as previously described.^15^ We observed broad, but relatively low, the expression of *IL1RAP* and *IL1R1* transcripts in several clusters (*Figure 1B* and *C*; Supplementary material online, *Figure S1A*). The IL-33 receptor *IL1RL1* was also broadly expressed, with higher levels being observed in cluster 13 (cKit-expressing mast cells), while *IL1RL2* that facilitates IL-36 signalling displayed low levels of expression in all clusters (*Figure 1C*; Supplementary material online, *Figure S1B* and *C*). Analysis of IL1RAP-related cytokines revealed higher levels of *IL1B* in clusters 5, 6, and 12 (CD68^+^ myeloid cells), whereas *IL33* was primarily found in clusters 8, 9, and 10 (smooth muscle cells, endothelial cells). *IL1A*, *IL36B*, and *IL36G* were expressed at lower levels throughout (*Figure 1D*; Supplementary material online, *Figure S1D–H*), and the expression of *IL36A* was not detected in this data set. To determine the presence of IL1RAP protein on circulating and plaque leucocytes, we stained PBMCs and digested human carotid artery plaques with an anti-IL1RAP-Alexa Fluor 647 (AF647) antibody or an isotype control-AF647 IgG and performed flow cytometric analysis. We detected IL1RAP on both myeloid cells and T cells, with the highest levels of IL1RAP expression on CD14^+^ myeloid cells in both plaques and PBMCs (*Figure 1E* and *F*; Supplementary material online, *Figure S1I* and *J*).

### 3.2 IL1RAP is present on leucocytes in murine plaques and adventitia

**Figure 2:**
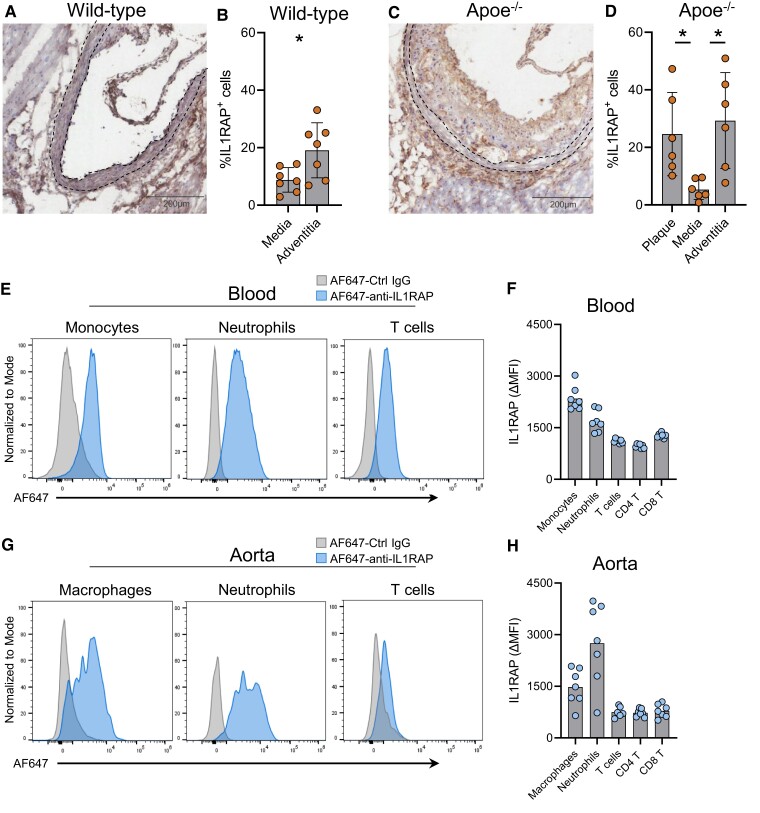
IL1RAP is present in atherosclerotic plaques of *Apoe^−/−^* mice. Immunohistochemical staining of IL1RAP and quantification of IL1RAP-expressing cells (per cent of total nucleated cells) in the aortic root of (*A* and *B*) wild-type (C57Bl/6, *n* = 7) and (*C* and *D*) hyperlipidaemic *Apoe^−/−^* mice (*n* = 6). The media layer is outlined by the dashed line. Flow cytometric analysis was performed to identify IL1RAP expression on leucocytes in the blood and digested atherosclerotic aortas of hyperlipidaemic *Apoe^−/−^* mice (*n* = 7). (*E*) Representative histograms of IL1RAP expression on monocytes (CD115^+^Ly6G^−^CD11b^+^), neutrophils (Ly6G^+^CD11b^+^), and T cells (CD11b^−^TCRβ^+^) in the blood and (*F*) quantification of IL1RAP MFI (ΔMFI) on leucocytes in the blood. (*G*) Representative histograms of IL1RAP expression on macrophages, neutrophils, and T cells in digested aorta and (*H*) quantification of IL1RAP (ΔMFI) on leucocytes in digested aorta. ΔMFI defined as the difference in MFI in each cell population between anti-IL1RAP and isotype antibody staining. Analysed with (*B*) Student’s unpaired *t*-test and (*D*) Kruskal–Wallis test with Dunn’s multiple comparisons test.

Next, we analysed murine aortic roots for the presence of IL1RAP by immunohistochemistry and immunofluorescence. Notably, IL1RAP staining of the adventitia was present in healthy control C57Bl/6 mice (*Figure 2A* and *B*), and we observed an increased density of IL1RAP^+^ cells in the adventitia compared with the media. In hyperlipidaemic *Apoe^−/−^* mice, we observed IL1RAP staining in both the plaque and the surrounding adventitia and to a lower extent in the media (*Figure 2C* and *D*; isotype staining shown in Supplementary material online, *Figure S2A*).

To validate these findings, we used flow cytometry to detect IL1RAP cell surface expression on circulating leucocytes as well as cells obtained by digestion of atherosclerotic aortas derived from hypercholesterolaemic *Apoe^−/−^* mice. In the blood, IL1RAP mean fluorescence intensity (MFI) was higher on blood monocytes and neutrophils compared with T cells (*Figure 2E* and *F*) in hyperlipidaemic *Apoe^−/−^* mice. Differentiating on blood monocyte subsets, we observed higher MFI levels of IL1RAP on patrolling Ly6C^lo^ monocytes (see Supplementary material online, *Figure S2B*). Likewise, aortic myeloid cells expressed higher levels of IL1RAP compared with T cells (*Figure 2G* and *H*) and co-localization of IL1RAP with CD68^+^ macrophages and CD3^+^ T cells was confirmed by immunofluorescence staining of murine aortic subvalvular plaques (see Supplementary material online, *Figure S2C*). IL1RAP expression was higher on the Ly6C^hi^ subset compared with the Ly6C^low^ subset of aortic CD11b^+^Ly6G^−^CD64^+^ monocyte/macrophages (see Supplementary material online, *Figure S2D*). Notably, expression level of IL1RAP on aortic neutrophils was, on average, increased by 70% compared with circulating neutrophils (circulating neutrophil average MFI: 1695, *Figure 2F*; aortic neutrophil average MFI: 2915, *Figure 2H*). Analysis of wild-type mice demonstrated similar patterns of IL1RAP expression, suggesting that the levels of IL1RAP expression on these cells are not contingent on hypercholesterolaemia (see Supplementary material online, *Figure S2E*).

### 3.3 Blockade of IL1RAP signalling limits plaque development

**Figure 3:**
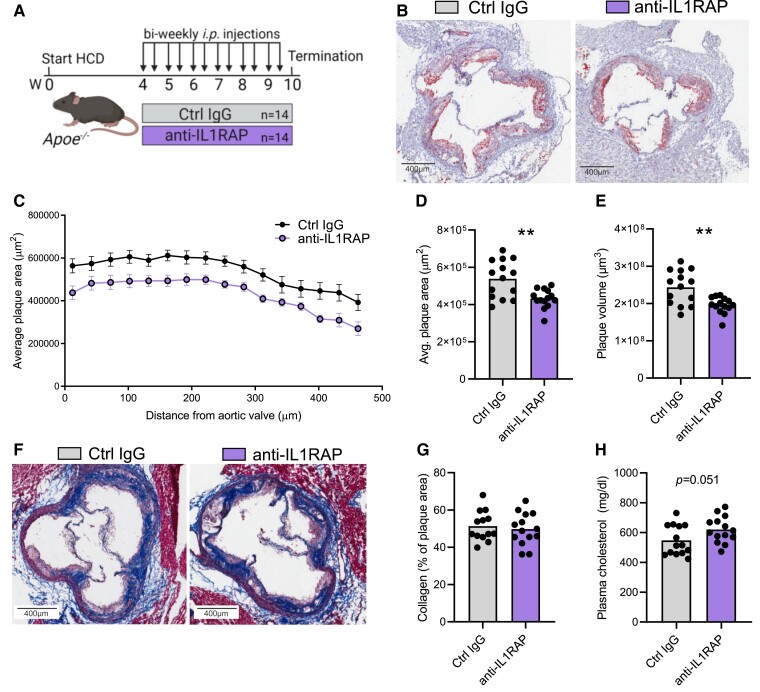
IL1RAP blockade reduces plaque burden. (*A*) Female *Apoe^−/−^* mice were treated biweekly with *i.p.* injections of either anti-IL1RAP antibody or control isotype IgG (Ctrl IgG) (*n* = 14/group) for 6 weeks, for a total of 10 weeks HCD. (*B*) Representative lipid staining (Oil Red O) of HCD-fed *Apoe*^−/−^ mice treated with anti-IL1RAP antibodies or isotype. (*C*) Quantification of average subvalvular plaque area progressing through the aortic valve. Dots denote average of all mice in each treatment group at indicated distance within the aortic valve. Quantification of (*D*) average plaque area (*P* = 0.0016) and (*E*) plaque volume (*P* = 0.0016). (*F*) Representative collagen staining (Masson’s trichrome) and (*G*) quantification of relative collagen area in aortic root plaques. (*H*) Total plasma cholesterol levels. Analysed with Student’s unpaired *t*-test.

To evaluate the effect of IL1RAP on the development of atherosclerosis, we administered a blocking non-depleting (LALA-PG modified) anti-IL1RAP antibody or isotype control IgG to *Apoe^−/−^* mice. Mice were fed HCD for 10 weeks and administered anti-IL1RAP or an isotype control IgG (*n* = 14/group) twice a week for the final 6 weeks of the study (*Figure 3A*). Analysis of subvalvular plaques in atherosclerotic mice revealed a significant 20% reduction in both plaque volume and average plaque area in mice treated with IL1RAP blockade (*Figure 3B–E*). Per cent plaque area stained for collagen was not affected (*Figure 3F* and *G*). Necrotic core area was similar comparing treatment groups (see Supplementary material online, *Figure S3A*).

The reduction in plaque size was not mediated by a reduction in cholesterol. A trend towards increased levels of total plasma cholesterol was observed in anti-IL1RAP-treated mice (*Figure 3H*), and body weights were unaffected (see Supplementary material online, *Figure S3B*). In a separate experiment, anti-IL1RAP treatment of C57Bl/6 wild-type mice did not impact cholesterol levels (see Supplementary material online, *Figure S3C*).

### 3.4 IL1RAP blockade reduces leucocyte populations in the atherosclerotic plaque

**Figure 4:**
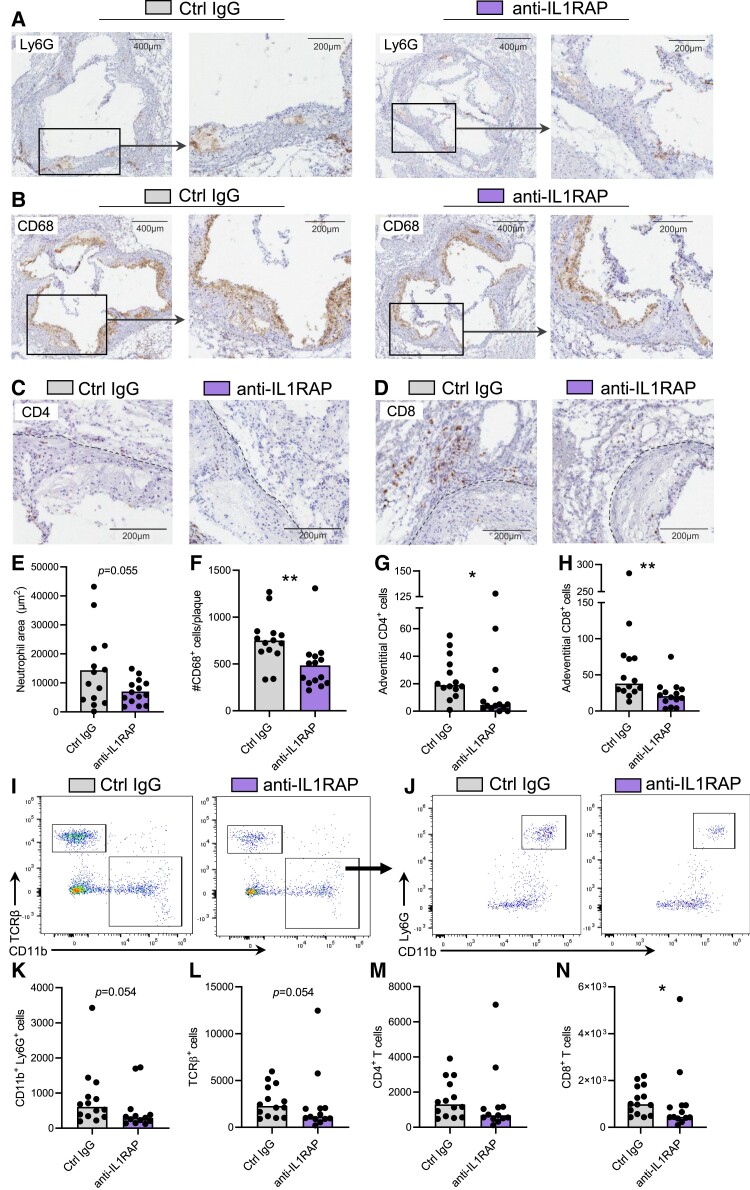
IL1RAP blockade limits leucocyte accumulation in the atherosclerotic plaque and adventitia. Representative immunohistochemical images of subvalvular aortic plaques of HCD-fed *Apoe*^−/−^ mice treated with anti-IL1RAP antibodies or isotype control (Ctrl IgG) (*n* = 14/group) stained for (*A*) neutrophils (Ly6G), (*B*) macrophages (CD68), (*C*) CD4^+^ T cells (CD4), and (*D*) CD8^+^ T cells (CD8). Quantification of (*E*) neutrophil area within the plaque and (*F*) number of CD68^+^ cells per plaque section (*P* = 0.0014). Quantification of adventitial (*G*) CD4^+^ T cells (*P* = 0.0248) and (*H*) CD8^+^ T cells (*P* = 0.0047). Full aortas from the same mice were enzymatically digested and single-cell suspensions were analysed via flow cytometry. (*I*–*J*) Representative flow cytometry plots and quantification of numbers of (*K*) neutrophils (CD11b^+^Ly6G^+^), (*L*) total T cells (CD11b^+^TCRβ^+^), (*M*) CD4^+^ T cells, and (*N*) CD8^+^ T cells (*P* = 0.0427) in digested aortas, as determined by flow cytometry. Analysed with Student’s unpaired *t*-test or Mann–Whitney *U* test.

To investigate how IL1RAP blockade affects the abundance of leucocytes in the atherosclerotic lesion, subvalvular plaques were stained for the presence of neutrophils (Ly6G), macrophages (CD68), and CD4^+^ and CD8^+^ T cells (*Figure 4A–D*). Treatment with anti-IL1RAP antibody led to a trend towards reduced levels of plaque neutrophils (*Figure 4E*; *P* = 0.055) and a significant reduction in plaque macrophages (*Figure 4F*). While we observed trends for reduced CD4^+^ plaque T cells (see Supplementary material online, *Figure S4A* and *B*), adventitial CD4^+^ and CD8^+^ T cells were both significantly reduced in anti-IL1RAP-treated mice compared with control mice (*Figure 4G* and *H*). Flow cytometric analysis of digested whole aortas (*Figure 4I* and *J*) displayed similar patterns, with trends towards reductions of numbers of aortic neutrophils (CD11b^+^Ly6G^+^) (*P* = 0.054; *Figure 4K*) and total TCRβ^+^ T cells (*P* = 0.054; *Figure 4L*). While numbers of aortic CD4^+^ T cells were not significantly lower in anti-IL1RAP-treated mice (*Figure 4M*), a significant reduction of aortic CD8^+^ T cells (*Figure 4N*) was observed. Taken together, blocking IL1RAP reduced the levels of leucocytes in the atherosclerotic aorta.

### 3.5 IL1RAP blockade limits hyperlipidaemia-driven haematopoiesis but does not affect circulating leucocyte populations

**Figure 5:**
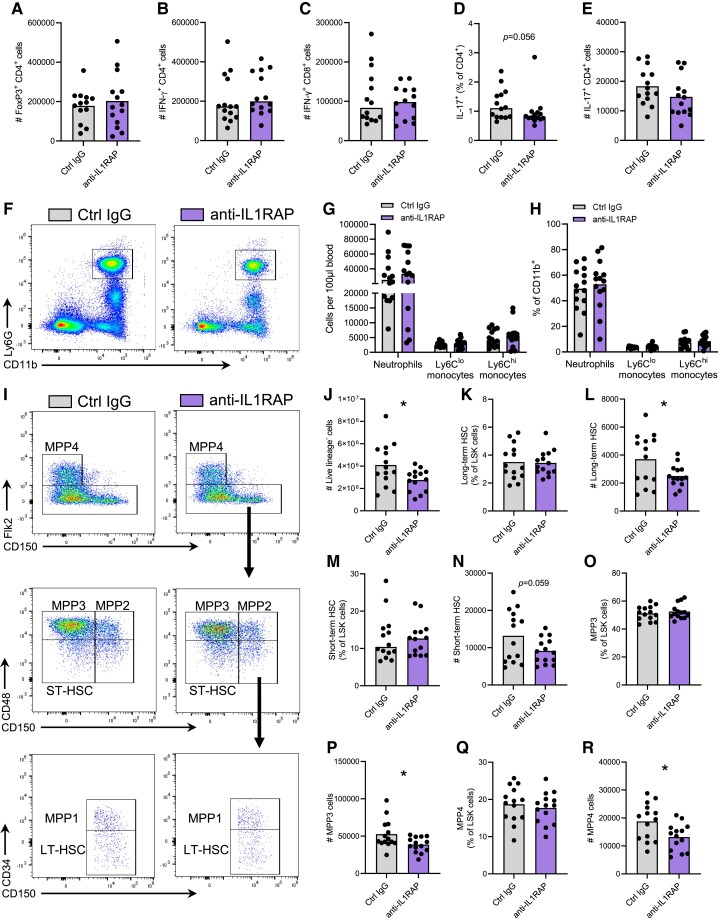
Systemic effects of IL1RAP blockade in *Apoe^−/−^* mice. Immune cell composition was analysed in spleen, blood, and bone marrow from HCD-fed *Apoe*^−/−^ mice treated with anti-IL1RAP antibodies or isotype control (Ctrl IgG) (*n* = 14/group). (*A*) Quantification of counts of regulatory T cells (FoxP3^+^CD4^+^) per spleen. Splenocytes were stimulated with PMA/ionomycin and Brefeldin A for 4 h to analyse cytokine production and T cell subsets. Quantification of counts of IFN-γ-producing (*B*) CD4^+^ T cells and (*C*) CD8^+^ T cells per spleen. Quantification of (*D*) counts per spleen and (*E*) proportion of IL-17-producing CD4^+^ T cells. (*F*) Representative flow cytometry plot of circulating neutrophils. Quantification of neutrophils (CD11b^+^Ly6G^+^), Ly6C^low^ monocytes (CD11b^+^Ly6G^−^CD115^+^), and Ly6C^high^ monocytes, both as (*G*) counts per 100 μL of blood and (*H*) per cent of CD11b^+^ myeloid cells. (*I*) Representative flow cytometry plots of LT-HSC, ST-HSC, and MPP populations in bone marrow. Quantification of (*J*) numbers of live lineage-negative cells (*P* = 0.0283). Percentages and counts of (*K* and *L*) LT-HSCs (*P* = 0.0323), (*M* and *N*) ST-HSCs, (*O* and *P*) MPP3 cells (*P =* 0.024), and (*Q* and *R*) MPP4 cells (*P =* 0.0156). Bone marrow population percentages given as per cent of LSK cells, counts given as per leg (one tibia and one femur). Analysed with Student’s unpaired *t*-test or Mann–Whitney *U* test.

Next, we studied how IL1RAP affected leucocyte composition in secondary lymphoid organs, blood, and bone marrow. We did not observe any changes in total CD4^+^ T cells and CD8^+^ T cells in spleen- and aortic-draining lymph nodes (see Supplementary material online, *Figure S5A* and *B*). Furthermore, no differences in splenic regulatory T cells (TCRβ^+^CD4^+^FoxP3^+^) (*Figure 5A*; Supplementary material online, *Figure S5C*) or ST2^+^ CD4^+^ T cells (see Supplementary material online, *Figure S5D* and *E*) were observed. Splenocytes were stimulated to enumerate T helper cell subsets based on their cytokine release. There was no change in the numbers (*Figure 5B* and *C*) or percentage (see Supplementary material online, *Figure S5F* and *G*) of interferon-γ-producing CD4^+^ or CD8^+^ T cells with anti-IL1RAP treatment, nor were there any changes in the numbers or percentage of IL-4-producing CD4^+^ or granzyme B-producing CD8^+^ T cells (see Supplementary material online, *Figure S5H–K*). IL-1 signalling has been shown to promote T helper 17 (Th17) polarization in atherosclerotic mice.^19^ We observed a trend towards reduced numbers and percentage of Th17 cells in spleens of anti-IL1RAP-treated mice (*Figure 5D* and *E*). Circulating neutrophils, monocytes (*Figure 5F–H*), and T cells (see Supplementary material online, *Figure S5L*) were not affected by IL1RAP blockade. We did not observe any differences in natural IgM levels against PC-reactive or Cu-oxLDL (see Supplementary material online, *Figure S5M* and *N*). In line with the limited effect of IL1RAP blockade on systemic immune composition, levels of chemokines and cytokines in plasma were not significantly changed (see Supplementary material online, *Figure S5O*).

It is well established that a HCD increases haematopoiesis in *Apoe^−/−^* mice,^20^ and IL-1 signalling has previously been shown to affect haematopoietic stem cells (HSCs) and myelopoiesis.^21,22^ Accordingly, we observed that IL1RAP blockade in HCD-fed *Apoe^−/−^* mice significantly decreased bone marrow cellularity and numbers of lineage-negative Sca1^+^cKit^+^ (LSK) HSCs, long-term HSC (LT-HSC), short-term HSC (ST-HSC), and multipotent progenitor (MPP) subsets 1–4 (*Figure 5I–R*; Supplementary material online, *Figure S5P–T*). However, we did not observe any changes in the proportions of HSC and MPP subsets relative to total LSK (*Figure 5I–R*; Supplementary material online, *Figure S5P–T*). These observations are in line with previous reports of IL-1β blockade limiting haematopoiesis in atherosclerotic mice.^22^

Studies of leukaemic cells suggest that IL1RAP may promote FLT3 and cKit signalling,^23^ with potential implications for haematopoiesis, dendritic cell homeostasis, and atherosclerosis.^24^ However, IL1RAP-deficient mice displayed normal haematopoiesis,^25^ arguing against a role for IL1RAP in modulating cKit signalling in non-leukaemic cells. To study any effects of anti-IL1RAP treatment on dendritic cell homeostasis, we performed a short-term experiment where a separate cohort of *Apoe^−/−^* mice were fed HCD for 2 weeks followed by biweekly injections of anti-IL1RAP or isotype control for 20 days (see Supplementary material online, *Figure S6A*). Flow cytometric analysis of aorta-draining iliac lymph nodes revealed no changes in either counts or percentages of CD11c^+^MHC-II^+^ dendritic cells nor change in DC subsets (CD11b^+/−^CD103^+/−^) or MFI of DC activation markers (MHC-II, CD40, CD80, and CD86; Supplementary material online, *Figure S6B–J*). Accordingly, levels of naïve, central memory, and effector memory CD4^+^ and CD8^+^ T cells were unaffected (see Supplementary material online, *Figure S6K* and *L*). These findings support the notion that dendritic cell homeostasis is not affected by anti-IL1RAP treatment.

### 3.6 Reduced expression of chemokines in anti-IL1RAP-treated mice

**Figure 6:**
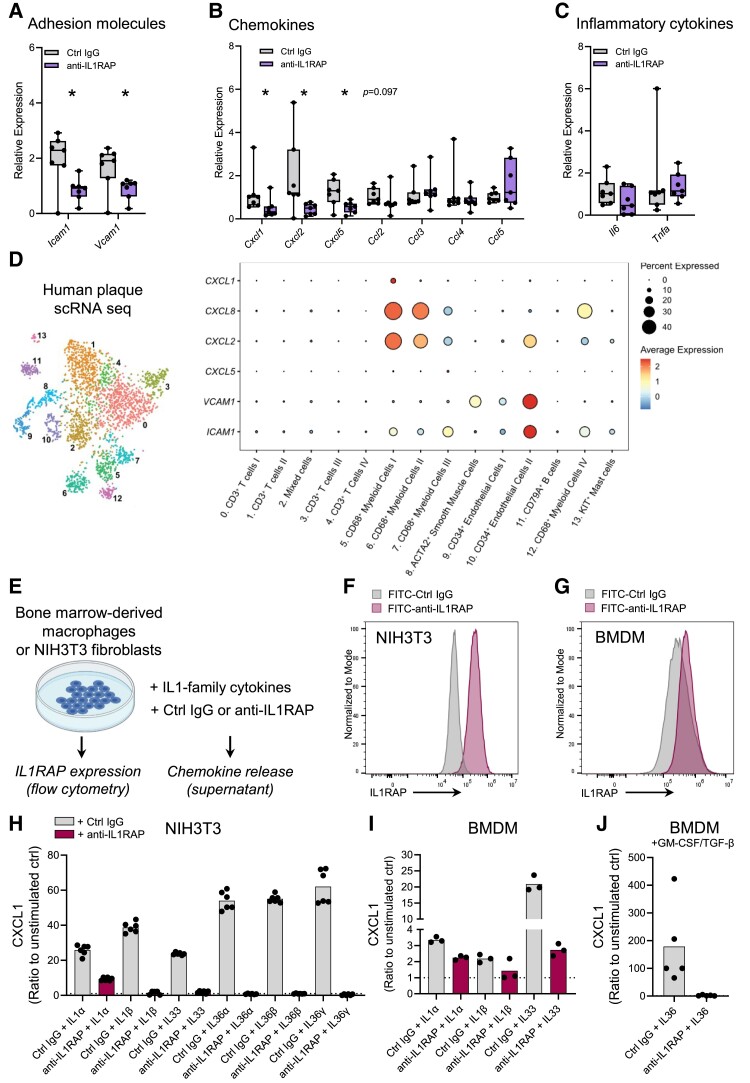
Reduced expression of chemokines in anti-IL1RAP-treated mice. RNA was isolated from BCAs from HCD-fed *Apoe*^−/−^ mice treated with anti-IL1RAP antibodies or isotype control (Ctrl IgG) (pooled samples, *n* = 2/pool; *n* = 7/group). Real-time PCR quantification of relative expression (2^−ΔΔCt^) of (*A*) adhesion molecules, (*B*) chemokines, and (*C*) inflammatory cytokines. (*D*) Dot plot of gene expression of chemokines and adhesion molecules in human carotid artery plaques. (*E*) BMDMs from wild-type (C57Bl/6) mice and a mouse fibroblast cell line (NIH3T3) were stimulated with either IL-1α, IL-1β, IL-33, or IL-36 in the presence of anti-IL1RAP antibody or isotype control (Ctrl IgG). (*F* and *G*) Expression of IL1RAP on BMDMs and NIH3T3 fibroblasts determined by flow cytometry. (*H*) Quantification of release of CXCL1 by NIH3T3 fibroblasts under each treatment condition (*n* = 6). Quantification of release of CXCL1 by (*I*) BMDMs (*n* = 3) stimulated with IL-1α, IL-1β, or IL-33 or (*J*) BMDMs (pre-treated with GM-CSF/TGF-β; *n* = 5) stimulated with IL-36 (IL-36α, IL36-β, and IL-36γ). (*A–C*) Bars denote median, analysed with Mann–Whitney *U* test (*P = 0.0262). (*H–J*) Bars denote mean.

We hypothesized that IL1RAP blockade limits chemokine production in the atherosclerotic plaque, leading to diminished leucocyte accumulation. To test if chemokines related to leucocyte recruitment were affected in mice treated with anti-IL1RAP antibodies, we quantified expression levels of inflammation-associated genes in pooled (*n* = 2/pool) BCAs of mice treated with anti-IL1RAP or isotype control IgG (*n* = 7/group). Gene expression levels for the adhesion molecules *Icam1* and *Vcam1*, as well as the CXCR2 ligands *Cxcl1*, *Cxcl2*, and *Cxcl5*, were reduced in BCAs of anti-IL1RAP-treated mice (*Figure 6A* and *B*). Relative expression levels for the chemokines *Ccl2*, *Ccl3*, *Ccl4*, and *Ccl5*, and inflammatory mediators *Tnfa* and *Il6*, were not significantly affected by IL1RAP blockade (*Figure 6B* and *C*).

We analysed single-cell RNA sequencing data of human carotid plaques for expression patterns of the genes that we observed to be affected by anti-IL1RAP treatment (*Figure 6D*; Supplementary material online, *Figure S7A–F*). In agreement with the important role of ICAM-1 and VCAM-1 on endothelial cells in recruitment of leucocytes, *ICAM1* and *VCAM1* gene expression was found at highest levels in CD34^+^ endothelial cells (*Figure 6D*). Of the chemokines we analysed CXCL8, a human homologue to CXCL1 in mice, and CXCL2 were found to be expressed at high levels clusters of CD68^+^ myeloid cells (*Figure 6D*), suggesting that myeloid cells are the key producers of these chemokines in human plaques.

Finally, to study if IL1RAP blockade can limit the release of chemokines *in vitro*, we stimulated bone marrow-derived macrophages (BMDMs) and a mouse fibroblast cell line (NIH3T3) with IL-1, IL-33, or IL-36 in the presence of anti-IL1RAP or isotype control IgG and measured release of cytokines in the cell culture supernatant (*Figure 6E*). Previous reports have shown that IL1RAP-related cytokines also target fibroblasts,^18,26,27^ and fibroblasts contribute to the inflammatory response as well as to infarct size and cardiac remodelling post-acute myocardial infarction.^28,29^ IL1RAP expression on NIH3T3 fibroblasts and BMDM before stimulation was detected by flow cytometry (*Figure 6F* and *G*). IL-1α, IL-1β, and IL-33 induced CXCL1 release by NIH3T3 fibroblasts, which could be abolished by blocking IL1RAP (*Figure 6H*). BMDM production of CXCL1 in response to IL-33 was abrogated by IL1RAP blockade; however, IL-1α or IL-1β did not induce robust CXCL1 release (*Figure 6I*). IL-36 induced a substantial CXCL1 release from NIH3T3 fibroblasts, which could be blocked by anti-IL1RAP (*Figure 6H*), but BMDMs generated by standard procedures did not produce CXCL1 in response to IL-36 (data not shown). Previous reports have demonstrated that the expression of the IL-36 receptor (IL-36R) and responsiveness to IL-36 is contingent on GM-CSF and TGF-β signalling in BMDMs.^18^ Macrophages pre-incubated with GM-CSF/TGF-β responded to a combination of IL-36α, IL-36β, and IL-36γ with release of CXCL1, and the levels of CXCL1 produced could be limited by IL1RAP blockade (*Figure 6J*). Collectively, these results demonstrate that IL1RAP blockade may regulate chemokine production of both haematopoietic and non-haematopoietic cells, with implications for leucocyte recruitment and the development of atherosclerosis.

## 4. Discussion

Cytokines play a central role in development of atherosclerosis.^1,2^ Our study demonstrates that blockade of IL1RAP, a signalling co-receptor of IL-1, IL-33, and IL-36 signalling, limits plaque burden as well as chemokine expression and myeloid cell accumulation in atherosclerotic *Apoe^−/−^* mice.

Several studies have demonstrated athero- and cardio-protective effects of treatment that target the IL-1 pathway. In mice, treatment with monoclonal anti-IL-1β has been shown to reduce plaque inflammation and lesion development.^5,22^ The CANTOS trial investigated the effect of canakinumab, a neutralizing anti-IL-1β monoclonal antibody, on adverse cardiovascular outcomes in high-risk patients. Although this study reported a significant reduction in adverse cardiovascular events in treated patients, it did not result in a difference in all-cause mortality.^4^ Another widely used therapeutic targeting IL-1 signalling is anakinra, a recombinant IL-1 receptor antagonist (IL-1Ra). Interfering with both IL-1α and IL-1β signalling, administration of anakinra has been shown to reduce plaque formation in *Apoe^−/−^* mice,^30,31^ as well as reduce systemic inflammation and heart failure-related deaths in patients post ST-segment-elevation myocardial infarction.^32^ Colchicine has been shown to interfere with the NLRP3 inflammasome, IL-1β maturation and release, and neutrophil trafficking.^33^ Treatment with colchicine in patients who experienced a recent myocardial infarction (COLCOT trial)^34^ or patients with chronic coronary disease (LoDoCo2 trial)^35^ led to a decrease in adverse cardiovascular events compared with placebo controls. While these studies cumulatively provide a proof-of-concept of IL-1 focused anti-inflammatory treatment reducing cardiovascular risk, there remains an unmet need for novel therapies.

Previous studies have demonstrated the presence of IL-1 family cytokines^12,36,37^ and their receptors^37,38^ in atherosclerotic plaques. We show that IL1RAP is present in murine atherosclerotic plaques and vascular adventitia and is highly expressed on neutrophils, monocytes, and macrophages. Flow cytometric analysis revealed high levels of IL1RAP on CD11b^+^CD14^+^ macrophages, suggesting that this population may be a key target for IL1RAP blockade in atherosclerosis. The cytokines that signal through IL1RAP elicit production of cytokines and chemokines associated with inflammation and leucocyte influx. In the present study, IL1RAP blockade led to reductions in levels of leucocytes in plaques paired with reductions in gene expression of *Vcam1*, *Icam1*, *Cxcl1* (also called GRO-α in humans or KC in mice), *Cxcl2* (also called GRO-β or MIP2a in humans), and *Cxcl5* (also called ENA-78 in humans or LIX in mice) in BCAs. We did not observe significant changes in *Il6* or *Tnfa* expression, indicating that these cytokines may be regulated by auxiliary pathways in the atherosclerotic plaque. Both ICAM-1 and VCAM-1 aid in the transmigration of leucocytes to sites of inflammation, and upregulation of VCAM1 and ICAM1 at lesion prone sites has been reported in mice and rabbits.^39,40^ Moreover, studies have shown reduced monocyte homing to atherosclerotic lesions,^41^ as well as reduced plaque size,^42^ in mice when interfering with VCAM-1 and ICAM-1 function. The observed reductions in plaque VCAM1 and ICAM1 mRNA expression suggest that anti-IL1RAP treatment may limit plaque development in part by reducing expression of adhesion molecules on endothelial cells and smooth muscle cells, leading to reduced recruitment of myeloid cells to the plaque.

CXCL1, CXCL2, and CXCL5 all share the common receptor CXCR2, which is expressed on neutrophils, monocytes, macrophages, and mast cells, and are involved in trafficking of these leucocytes under homeostatic conditions as well as during inflammation.^43^ CXCL1 and CXCL2 have been shown to regulate inflammasome activation in macrophages, and several CXC-chemokines have been implicated in cardiovascular disease.^44,45^ The role of CXCL1 promoting leucocyte influx in atherosclerosis has been corroborated by several previous reports.^46–48^ We demonstrate by analysis of single-cell gene expression of human plaque leucocytes that CXCL2 and CXCL8, the functional human homologue of murine CXCL1, were mainly expressed in CD68^+^ myeloid cells. Altogether, these findings suggest that IL1RAP signalling in plaque macrophages or other cells such as endothelial cells promotes local inflammation by inducing chemokine release, leading to a vicious cycle of myeloid cell recruitment. In addition, the observation that blockade of IL1RAP *in vitro* inhibits cytokine-induced release of CXCL1 in fibroblasts suggests that administration of anti-IL1RAP may also temper chemokine production in non-haematopoietic cells residing in the plaque or adventitia.^49,50^ This is in accordance with previous reports of IL1RAP family cytokines promoting chemokine release of haematopoietic and non-haematopoietic cells.^37,51–53^ Our results suggest that fibroblasts are relatively more efficient CXCL1 producers in response to IL-1α and IL-1β compared with BMDMs *in vitro*. However, whether human plaque macrophages are more sensitive to IL-33 relative to IL-1α/IL-1β stimulation *in vivo* remains to be determined. We propose that blocking IL1RAP signalling, rather than an individual cytokine, may be more efficient in limiting leucocyte recruitment to the plaque and contributing to plaque stability in patients with elevated risk for cardiovascular disease.

IL-1 has repeatedly been shown to affect myelopoiesis.^21^ Blockade of IL-1β or NLRP3 inflammasome limits myelopoiesis in atherosclerotic mice,^22^ and IL-1β blockade limits myelopoiesis after myocardial infarction.^54^ In line with these previous reports, we observed reduced numbers of HSCs and multipotent progenitors in the bone marrow of mice treated with anti-IL1RAP. Despite reductions in the numbers of haematopoietic precursors, anti-IL1RAP treatment did not translate to changes in circulating monocytes or neutrophils. Supporting a limited effect on haematopoiesis and circulating leucocytes by IL1RAP blockade, mice deficient in IL1RAP displayed normal haematopoiesis.^25^ In line with limited systemic effects on anti-IL1RAP on myeloid cells, we did not observe any changes in plasma cytokine and chemokine levels.

T cells express receptors for IL1RAP family cytokines and are thus subject to immunomodulation by anti-IL1RAP. While we did not observe significantly reduced levels of T cells in plaques of anti-IL1RAP-treated mice, the density of both CD4^+^ and CD8^+^ T cells was reduced in the adventitia. How adventitial T cells relate to the progression of atherosclerosis is not well understood, but it is likely that the accumulation of these cells correlate to levels of plaque inflammation, as advanced atherosclerosis has been shown to promote generation of tertiary lymphoid structures in the adventitia.^55^ We propose that the reductions in adventitial T cells are secondary to reductions in the expression of chemokines and adhesion molecules which drive influx of leucocytes to the atherosclerotic artery. We have previously reported that IL-1R signalling in T cells affects Th17 polarization in atherosclerotic mice.^19^ Accordingly, we here observed trends towards reductions in levels of Th17 cells in spleen after anti-IL1RAP treatment, likely due to reductions in T cell IL-1R signalling. Although levels of T helper cells, ST2-expressing regulatory T cells, cytotoxic T cells, and serum cytokine profile were not affected by IL1RAP blockade, we cannot exclude local effects on T cells as mediating part of the reduced plaque burden observed after anti-IL1RAP treatment.

Effects on cholesterol levels cannot explain the reduction in plaque size by anti-IL1RAP treatment, as we observed a trend towards increased levels of total plasma cholesterol in anti-IL1RAP-treated mice. There are some reports in the literature on IL1RAP-dependent signalling modulating lipid levels. Mice overexpressing IL-1Ra exhibited a trend of increasing plasma cholesterol,^56^ and in an *in vitro* model of *Mycobacterium tuberculosis*, IL-36 deficiency was reported to increase cholesterol biosynthesis and LXR activation.^57^ Single-nucleotide polymorphisms associated with elevated expression of the soluble IL-1Ra, which acts as a decoy receptor for IL-1α and IL-1β, were associated with increasing levels of LDL cholesterol and coronary heart disease.^58^ This is in line with observations that patients treated with a soluble decoy receptor for IL-1 (rilonacept) for recurrent pericarditis exhibited modestly elevated levels of non-fasting LDL.^59^ However, in other studies, blocking IL-1α and IL-1β in combination did not affect cholesterol levels.^5,60^ In addition, no significant effects on cholesterol levels were observed in patients after anti-IL-1β blockade in the CANTOS study.^4^ Importantly, administration of anti-IL1RAP antibodies to normolipidaemic wild-type (C57Bl/6) mice in our study did not affect cholesterol levels compared with isotype controls. Taken together, it is possible that any potential influence on cholesterol levels from impaired IL-1 or IL-36 signalling after anti-IL1RAP antibody treatment is exacerbated in the *Apoe^−/−^* mouse, but there is little evidence that blocking IL-1 have these effects in humans.

Despite the majority of IL1RAP-related cytokines being associated with plaque burden and inflammation, there are also reports of atheroprotective effects of some of these cytokines. Treatment with IL-33 promotes expansion of type 2 innate lymphoid cells (ILC2) and increases levels of natural IgM, altogether limiting development of atherosclerotic plaques.^10^ However, a separate study did not find any differences in lesion size in neither IL-33 nor ST2 knockouts on an *Apoe^−/−^* background compared with controls.^9^ ILC2s are expanded in response to IL-33 and IL-2, and several reports have demonstrated that expansion of ILC2 limits atherosclerosis^61,62^ and promotes recovery after myocardial infarction.^63^ Although it is possible that our treatment limits levels of ILC2s in plaques and adventitia by blocking IL-33 signalling, ILC2s make up a small fraction of plaque leucocytes and are likely not the main drivers of pathogenesis of atherosclerosis. Though the majority of studies have demonstrated a pro-atherosclerotic role for IL-1β, there are reports of IL-1β promoting plaque stability and protective outward remodelling of the artery.^38^ Our results indicate that limiting the potential atheroprotective features ascribed to IL-33,^10^ or even IL-1β,^38^ is not, in our hands, able to overcome the benefit of blocking detrimental IL1RAP signalling.

We propose that IL-1α, IL-1β, IL-33, and IL-36α/β/y may all play a role at different stages of plaque development and in different patient categories. For example, IL-36 signalling has been implicated in several autoimmune diseases, such as rheumatoid arthritis, psoriasis, and psoriatic arthritis,^64^ all of which are related to an elevated risk of cardiovascular disease.^65^ IL-33 signalling has been implicated in heart failure and hypertension.^66^ Limiting IL1RAP signalling by anti-IL1RAP administration rather than blockade of individual cytokine may provide greater cardiovascular benefit than individual cytokine blockade. Our study design did not allow us to define the relative magnitude of each individual IL1RAP family cytokine on atherosclerosis. Given the evidence from the canakinumab trial, we propose that the reduced plaque burden observed after IL1RAP blockade is at least in part mediated by limiting IL-1 signalling but that IL-33 and IL-36 signalling may also act to aggravate atherosclerosis by promoting chemokine release. The relative atherogenic contribution of each of these individual cytokines is difficult to gauge but is likely to vary between patients. An additional limitation of our study is that all mechanistic studies were conducted in atherosclerotic *Apoe^−/−^* mice. Further, we restricted our analysis to only female mice, since, compared with their male counterparts, female *Apoe*^−/−^ mice develop atherosclerosis at an accelerated rate when given HCD. Thus, we did not evaluate potential sex-specific effects of IL1RAP blockade. Furthermore, anti-IL1RAP treatment was given between Week 4 and Week 10 of HCD feeding, and thus, we did not test the role of IL1RAP on early lesion formation. The rationale for this design was to better mimic the clinical situation, in which immunomodulatory treatments are likely to be administered to patients with already established atherosclerotic lesions.

A humanized anti-IL1RAP monoclonal antibody (CAN10, Cantargia AB), which also blocks IL-1, IL-33, and IL-36 signalling without interacting with Fc receptors (LALA mutated), is currently being evaluated in a phase I trial (NCT06143371). Another monoclonal anti-IL1RAP antibody (Nadunolimab, CAN04, Cantargia AB) that mainly blocks IL-1 signalling and has enhanced antibody-dependent cellular cytotoxicity (ADCC)-inducing properties (afucosylated) is currently undergoing clinical trials for efficacy against non–small-cell lung cancer, triple negative breast cancer, and pancreatic cancer in combination with chemotherapy (NCT05116891, NCT04990037, NCT03267316, NCT05181462) or pembrolizumab (anti-PD1; NCT04452214). In a phase I trial, nadunolimab as monotherapy treatment led to reductions in the levels of the cardiovascular risk factors CRP and IL-6, further arguing in favour of a cardiovascular benefit of IL1RAP blockade.^67^ Similar to canakinumab, administration of nadunolimab is associated with reduced levels of neutrophils.^67^ Phase I studies of CAN10, which does not promote ADCC, could potentially provide insights into its effects on inflammatory biomarkers relevant to cardiovascular disease as well as rates of adverse events.

In summary, our study demonstrates that limiting IL-1, IL-33, and IL-36 signalling by blocking IL1RAP reduces plaque inflammation and plaque burden in atherosclerotic mice. Our study highlights the potential benefits of targeting the IL1RAP-dependent cytokine pathways in patients with cardiovascular disease.

## Supplementary Material

cvae046_Supplementary_Data

## Data Availability

The data underlying this article will be shared on reasonable request to the corresponding author.
