# Supplementary material for: Interleukin-1 receptor accessory protein blockade limits the development of atherosclerosis and reduces plaque inflammation
Source: Cardiovasc Res. 2024 Apr 2;120(6):581–95. doi: 10.1093/cvr/cvae046 (PMC11074796; doi:10.1093/cvr/cvae046)
Supplement: cvae046_Supplementary_Data [file cvae046_supplementary_data.zip › SUPPL FIGURES_Mulholland et al_IL1RAP_2nd resubmission.pdf]

SUPPLEMENTAL FIGURES
Mulholland et al

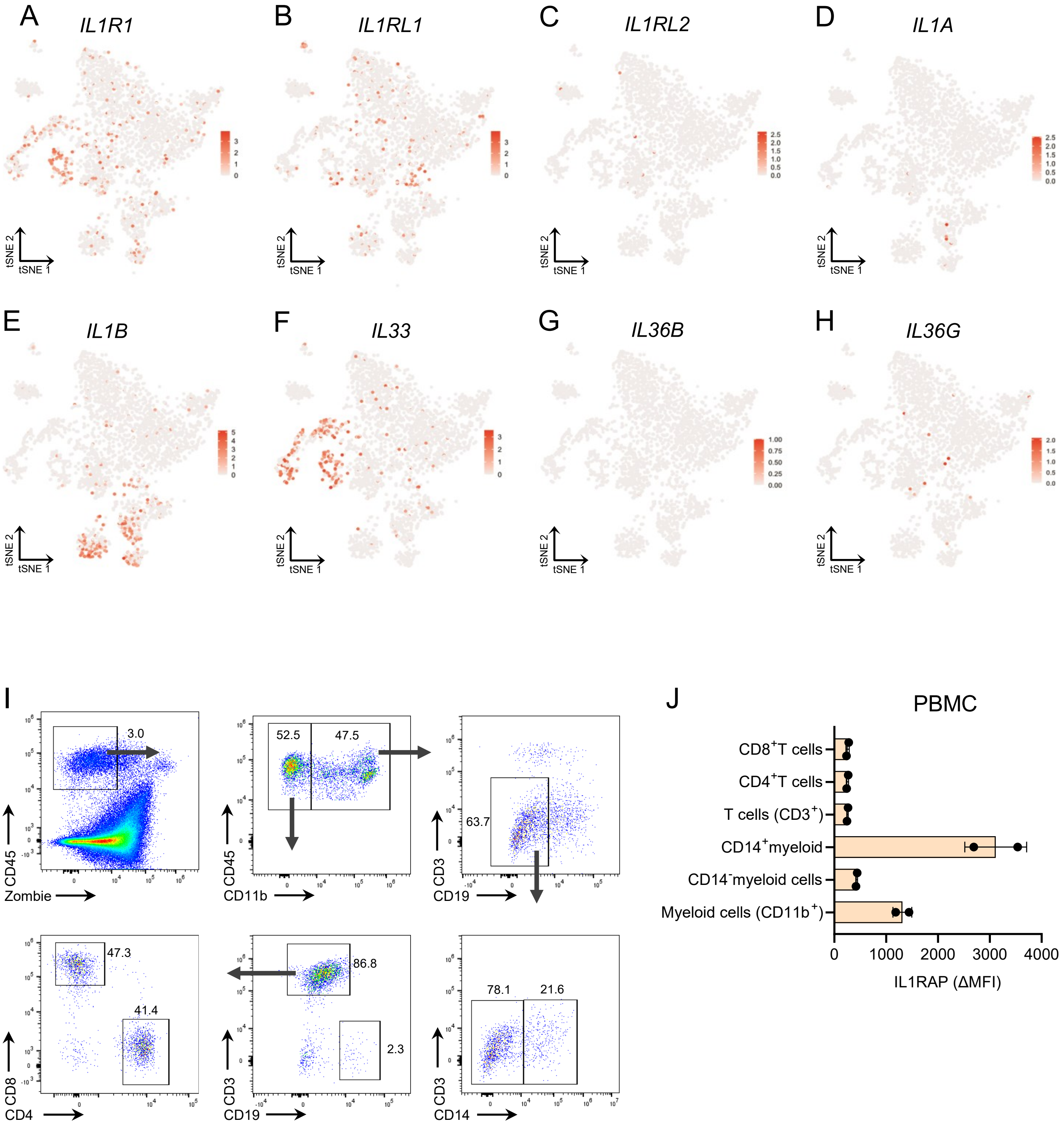

Supplemental Figure 1. Analysis of IL1RAP family cytokines and receptors in human carotid plaques.

Single-cell RNA sequencing data set of human carotid plaques (n=18) from patients that underwent carotid endarterectomy was analysed. Feature plots of gene expression of (A) *IL1R1,* (B) *IL1RL1,* (C) *IL1RL2,* (D) *IL1A,* (E) *IL1B,* (F) *IL33,* (G) *IL36B,* and (H) *IL36G*. (I) Gating strategy to identify cell populations in carotid plaque digests subsequently analysed for IL1RAP expression. (J) Levels of IL1RAP (ΔMFI) of leukocyte subpopulations in peripheral blood mononuclear cells (n=2).

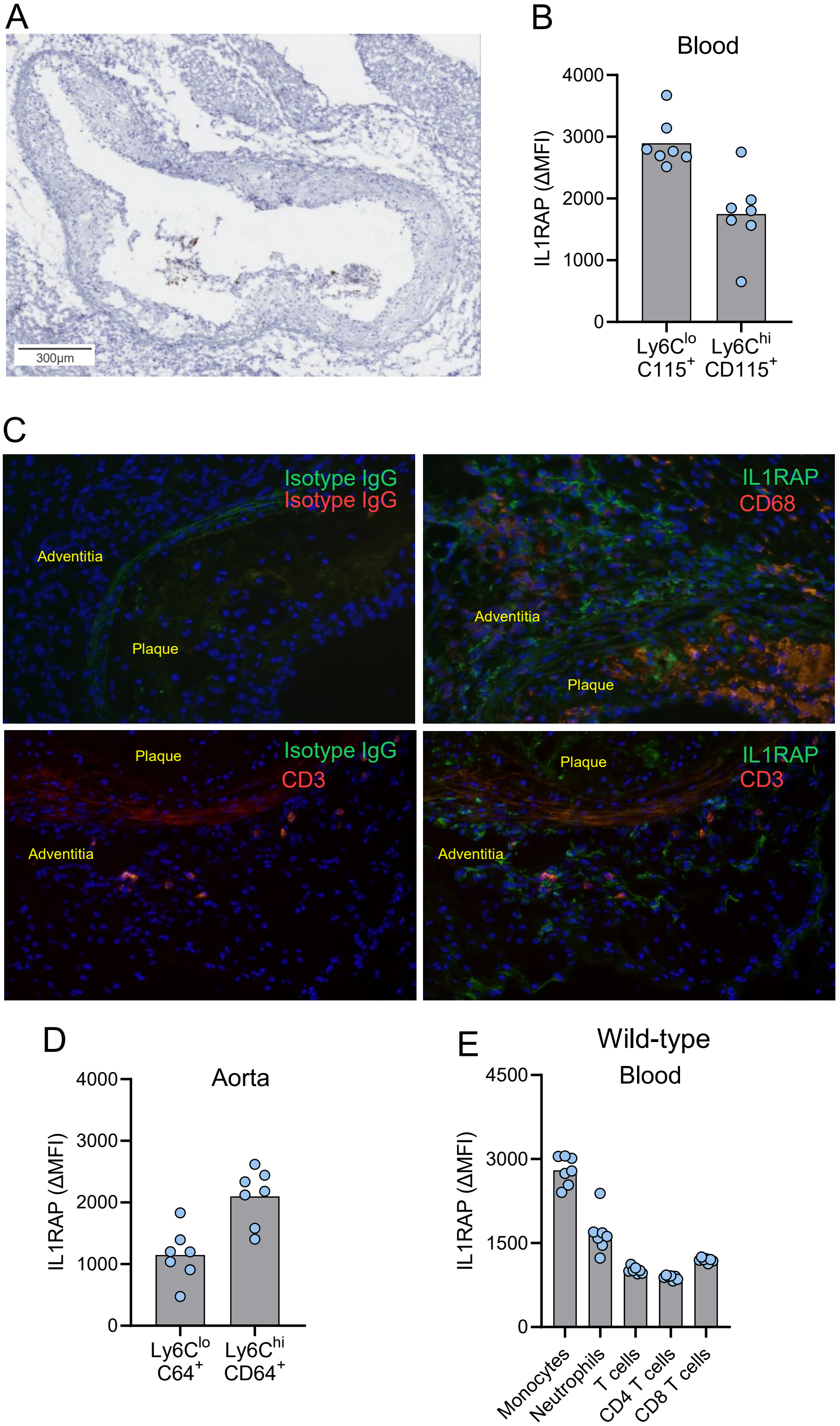

Supplemental Figure 2. Histological analysis of IL1RAP in murine plaques

(A) Representative isotype control immunohistochemical staining of subvalvular plaque of a high-cholesterol diet-fed *Apoe*$^{-/-}$ mouse. (B) IL1RAP mean fluorescence intensity (ΔMFI) on circulating Ly6C^{low} and Ly6C^{high} monocytes (CD115^{+}Ly6G^{-}CD11b^{+}) in *Apoe*$^{-/-}$ mice (n=7), analysed by flow cytometry. (C) Representative immunofluorescence images of co-staining for IL1RAP and CD68 or IL1RAP and CD3 in aortic subvalvular cross-sections in high-cholesterol diet-fed *Apoe*$^{-/-}$ mouse. (D) IL1RAP ΔMFI on Ly6C^{low} and Ly6C^{high} monocyte/macrophages (CD64^{+}Ly6G^{-}CD11b^{+}) in digested aortas of *Apoe*$^{-/-}$ mice (n=7), analysed by flow cytometry. (E) IL1RAP mean fluorescence intensity (ΔMFI) on leukocytes in blood on monocytes (CD115^{+}Ly6G^{-}CD11b^{+}), neutrophils (Ly6G^{+}CD11b^{+}), and T cells (CD11b^{-}TCRβ^{+}) in blood of wild-type (C57Bl/6) mice (n=7). ΔMFI defined as the difference in MFI in each cell population between anti-IL1RAP and isotype antibody staining; bars denote mean.

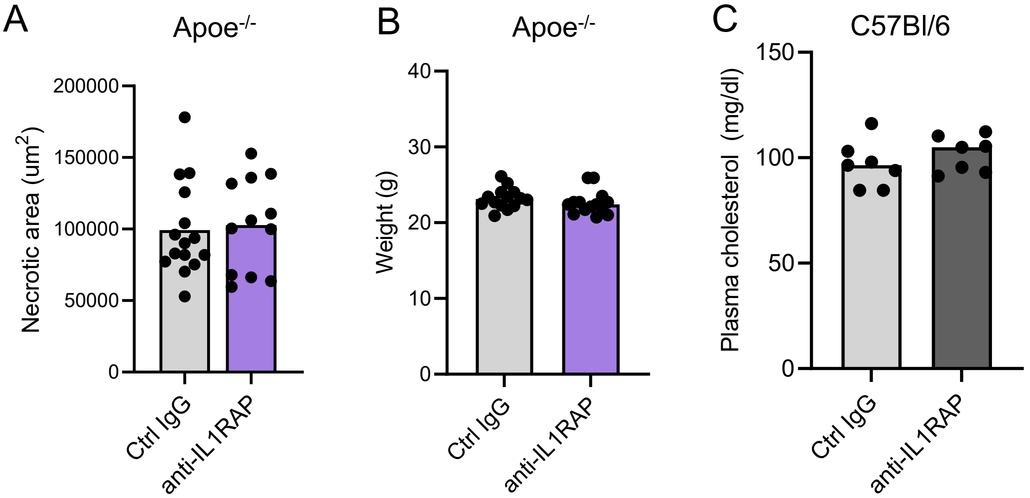

Supplemental Figure 3. In vivo blockade of IL1RAP

Female *Apoe*$^{-/-}$ mice were fed a high-cholesterol diet for a total of 10 weeks and treated biweekly with *i.p.* injections of either anti-IL1RAP antibody or control isotype IgG (Ctrl IgG) for the final six weeks (n=14/group). (A) Body weight of mice. In a separate experiment, wild-type (C57Bl/6) mice were kept on a normolipidaemic chow diet and administered anti-IL1RAP or isotype control IgG antibodies (Ctrl IgG, n=7/group). (B) Quantification of total plasma cholesterol. Analysed with Mann-Whitney's *U* test.

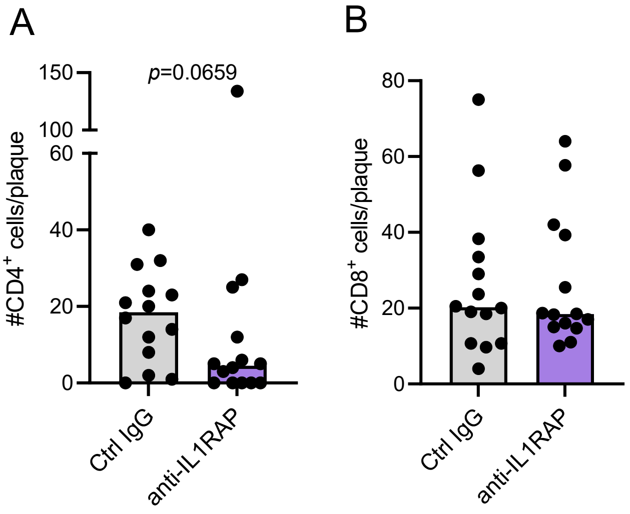

Supplemental Figure 4. Immunohistochemical analysis of T cells in murine atherosclerotic plaques

Immunohistochemical staining for CD4 and CD8 was performed on subvalvular aortic plaques of high-cholesterol diet-fed *Apoe*$^{-/-}$ mice treated with anti-IL1RAP antibodies or isotype control (Ctrl IgG, n=14/group). Quantification of total counts of (A) CD4^{+} T cells (CD4) and (B) CD8^{+} T cells (CD8) per plaque section. Analysed with Mann-Whitney's *U* test.

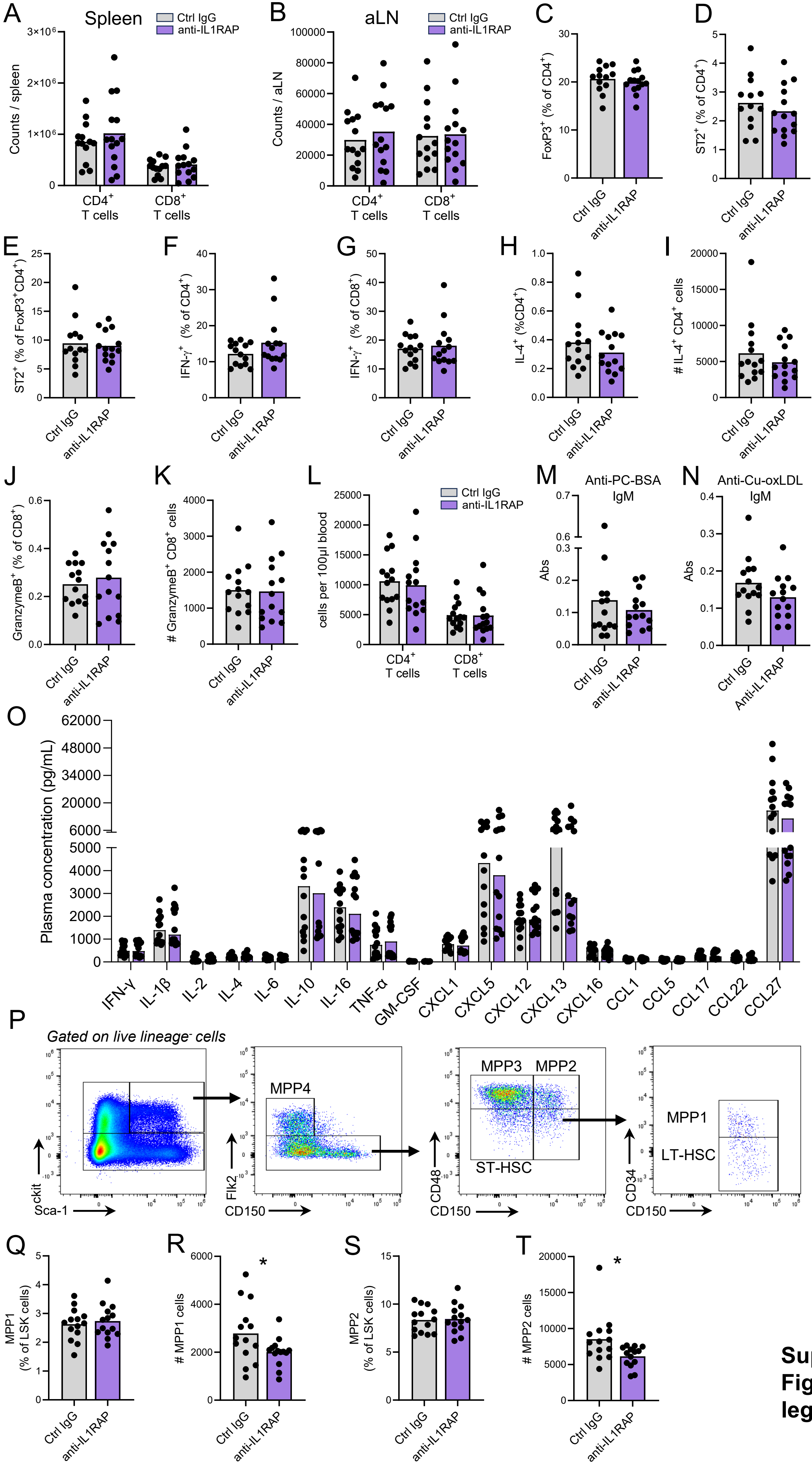

Supplemental Figure 5 legend →

Supplemental Figure 5. Analysis of immune composition and haematopoiesis in mice receiving anti-IL1RAP treatment

Immune cell composition was analysed in spleen, blood, and bone marrow from high-cholesterol diet-fed *Apoe*$^{-/-}$ mice treated with anti-IL1RAP antibodies or isotype control (Ctrl IgG, n=14/group). Total CD4^{+} and CD8^{+} T cell counts in (A) spleen and (B) aortic-draining lymph nodes. (C) Percent regulatory T cells (FoxP3^{+}CD4^{+}) in spleen. Percent of ST2 expression on (D) total CD4^{+} T cells and on (E) FoxP3^{+} regulatory T cells in spleen. Splenocytes were stimulated with PMA/ionomycin + Brefeldin A for 4 hours to analyse cytokine production and T cell subsets. Percent of splenic IFN-γ-producing (F) CD4^{+} T cells and (G) CD8^{+} T cells; (H) percent and (I) counts of IL-4-producing CD4+ T cells; (J) percent and (K) counts of granzyme B-producing CD8^{+} T cells. (L) Counts of circulating CD4^{+} and CD8^{+} T cells per 100 µl blood. Quantification of natural IgM antibodies against (M) phosphorylcholine-conjugated bovine serum albumin (PC-BSA) or (N) copper-oxidized low-density lipoprotein (Cu-oxLDL, pooled plasma samples). (O) Multiplex analysis of plasma cytokines and chemokines. (P) Gating strategy of bone marrow lineage-Sca1^{+}ckit^{+} (LSK) cells, long-term hematopoietic stem cells (LT-HSC), short-term HSC (ST-HSC), and multipotent progenitors (MPP) populations. Quantification of (Q) percent and (R) numbers of MMP1 cells (p=0.046). Quantification of (S) percent and (T) numbers of MMP2 cells (p=0.019). Bone marrow population percentages given as percent of LSK cells, counts given per leg (one tibia and one femur). Analysed with students unpaired t test or Mann-Whitney U test.

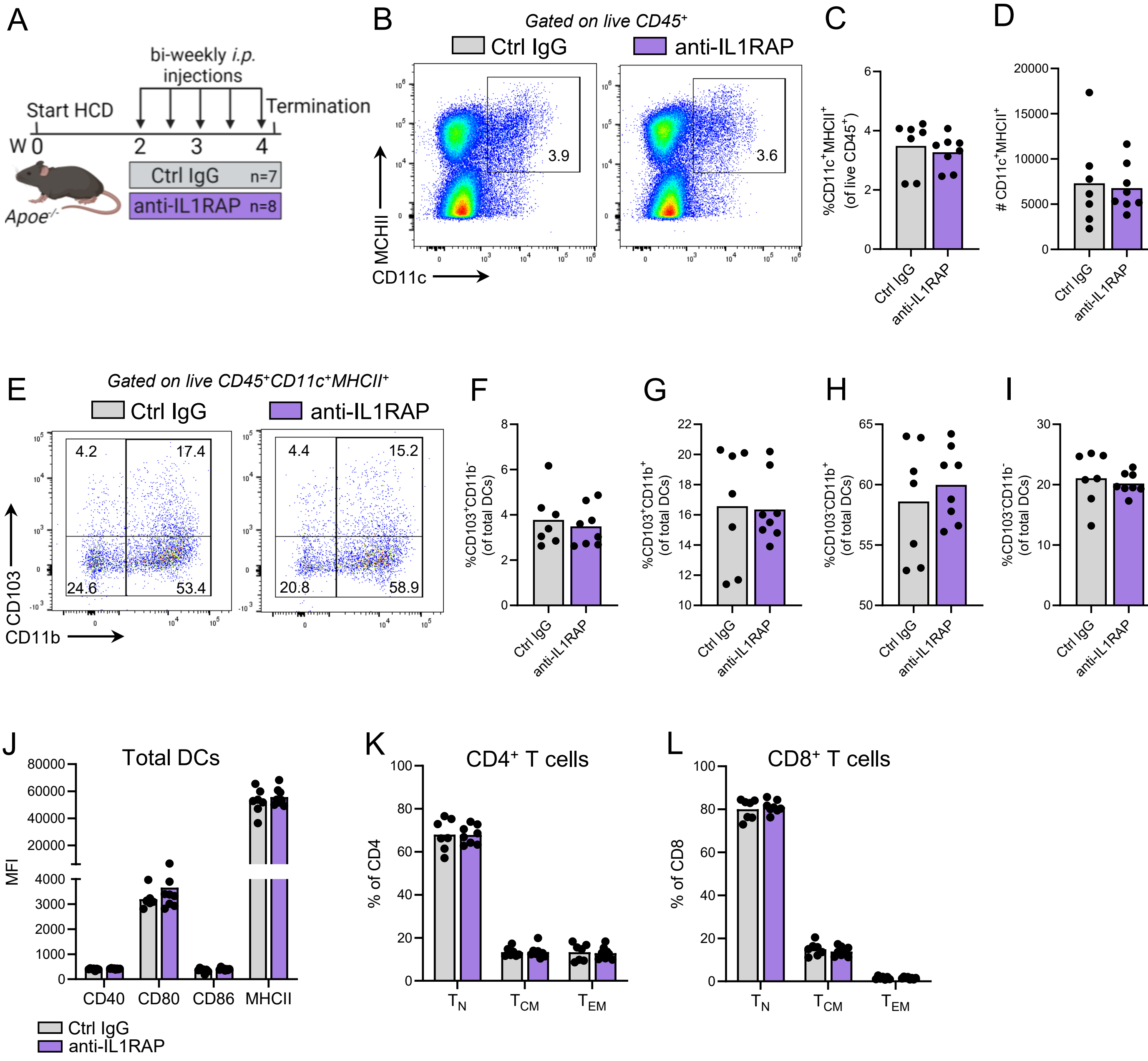

Supplemental Figure 6. Dendritic cell and T cell phenotype after short-term IL1RAP blockade of *Apoe*$^{-/-}$ mice

(A) Female *Apoe*$^{-/-}$ mice were treated biweekly with *i.p.* injections of either anti-IL1RAP antibody or control isotype IgG (Ctrl IgG) (n=7-8/group) for two weeks, for a total of four weeks high-cholesterol diet (HCD). Aorta-draining iliac lymph nodes were analysed via flow cytometry after termination. (B) Representative flow cytometry plots of total dendritic cells (DCs; MHCII^{+}CD11c^{+}). Quantification of (C) percent and (D) counts DCs. (E-I) Representative flow cytometry plots and quantification of DC subsets separated by CD11b and CD103 expression. (J) Expression (MFI) of activation markers on total DCs (MHCII^{+}CD11c^{+}). Distribution of memory phenotypes of (K) CD4^{+} and (L) CD8^{+} T cells (naïve, TN: CD62L^{+}CD44^{-}; central memory, TCM: CD62L^{+}CD44^{+}; effector memory, TEM: CD62L^{-}CD44^{+}).

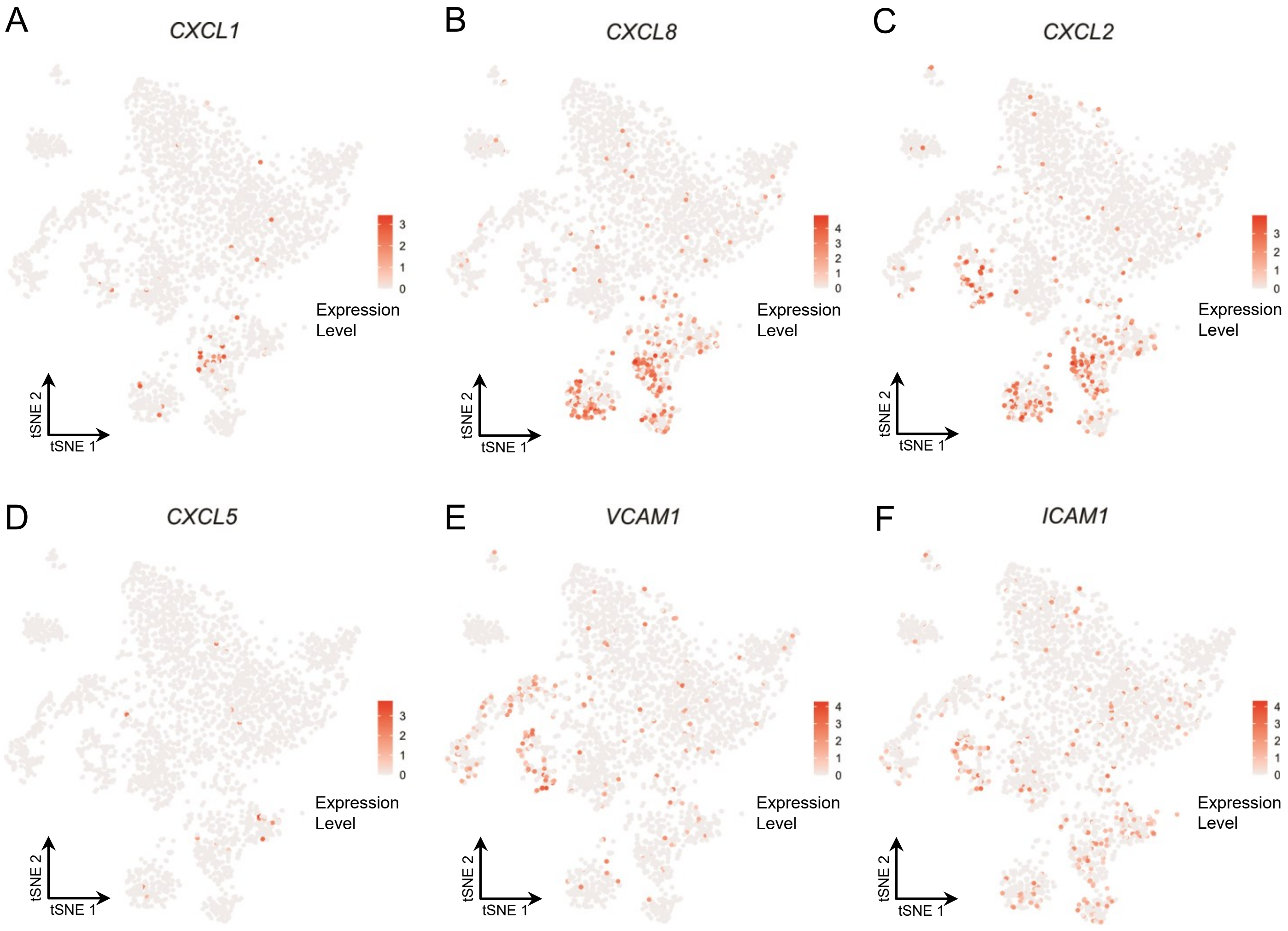

Supplemental Figure 7. Chemokine and adhesion molecule gene expression in human carotid plaques

Single-cell RNA sequencing of cells derived from human carotid plaques. Feature plots of gene expression of (A) *CXCL1,* (B) *CXCL8,* (C) *CXCL2*, (D) *CXCL5*, (E) *ICAM1*, and (F) *VCAM1*.
