# Supplementary material for: Interleukin-1 receptor accessory protein blockade limits the development of atherosclerosis and reduces plaque inflammation
Source: Cardiovasc Res. 2024 Apr 2;120(6):581–95. doi: 10.1093/cvr/cvae046 (PMC11074796; doi:10.1093/cvr/cvae046)
Supplement: cvae046_Supplementary_Data [file cvae046_supplementary_data.zip › Supplemental Methods_IL1RAP_2nd resubmission.pdf]

Supplemental Methods

Murine flow cytometry. The following fluorescent-conjugated antibodies were used in flow cytometry analysis, purchased from BioLegend (unless otherwise stated): CD44-AF488 (IM7), Ly6G-PE (1A8), TCRβ-PE/Dazzle (H57-597), CD8α-PerCP/Cy5.5 (53-6.7), CD11b-PE/Cy7 (M1/70), CD64-APC (X54-5/7.1), Ly6C-AF700 (HK1.4), CD4-APCCy7 (GK1.5), CD45.2-PB (104), CD115-APC (AFS98), NK1.1-PE/Cy7 (PK136), IFNγ-FITC (XMG1.2), IL17-APC (TC11-18H10.1), IL4-PE (11B11), CD64-PE (X54-5/7.1), CD115-PE (ASF98), Ly6C-PerCP/Cy5.5 (HK1.4), CD8α-AF700 (53-6.7), CD115-AF488 (AFS98), B220-PE (RA3-6B2), Ter119-PE (TER-119), CD11b-PE (M1/70), NK1.1-PE (PK136), CD19-PE (HIB19), CD3-PE (145-2C11), CD127-PE (A7R34), cKit-PerCP/Cy5.5 (2B8), CD150-PE/Cy7 (TC15-12F12.2), CD16/32-APC (93), CD48-APC/Cy7 (HM48-1), Sca1-BV421 (D7), CD16/32-(93), Zombie Aqua Live/Dead (423102), CD34-AF700 (RAM34, Invitrogen), Granzyme B-eFluor450 (NGZB, Invitrogen), CD135-PE/CF594 (A2F10.1, BD Bioscience), IL1RAP-AF647 (15F12, Cantargia AB) and isotype control-AF647 (Cantargia AB).

Human flow cytometry. The following dyes/antibodies were used for staining human plaques and peripheral blood mononuclear cells: Fixable Viability dye (eBioscience) and anti-CD45 (clone 2D1, Biolegend) anti-CD3 (clone OKT3, Biolegend) anti-CD4 (clone OKT4, eBioscience), anti-CD8 (clone SK1, Invitrogen), anti-CD11b (clone LM2, Biolegend), CD14 (clone 61D3, Biolegend) and anti-CD19 (clone HIB10, Biolegend).

RNA expression analysis. The following Taqman probes used for real-time PCR analysis: VCAM1 (Mm01320970_m1), ICAM1 (Mm00516023_m1), CXCL1 (Mm04207460_m1), CXCL2 (Mm00436450_m1), CXCL5 (Mm00436451_g1), CCL2 (Mm00441242_m1), CCL3 (Mm00441259_g1), CCL4 (Mm00443111_m1), CCL5 (Mm01302427_m1), IL6: (Mm00446190_m1), TNFα (Mm00443258_m1), 18S;R (Mm03928990_g1).

Immunohistochemistry and immunofluorescence Analysis of cellular composition of plaques was performed using the following antibodies: neutrophils, stained with anti-Ly6G (0.5 µg/mL in 5% rabbit serum; BioRad) as primary antibody and biotinylated rabbit anti-rat (1:1000 in TBS; Vector Laboratories) as secondary antibody; macrophages, stained with anti-CD68 (0.125 µg/mL in 5% rabbit serum; BioRad) as primary antibody and biotinylated rabbit anti-rat (1:1000 in TBS; Vector Laboratories) as secondary antibody; $CD4^+$ T cells, stained with anti-CD4 (0.625 µg/mL in 5% rabbit serum; BioRad) as primary antibody and biotinylated rabbit anti-rat (1:1000 in TBS; Vector Laboratories) as secondary antibody; $CD8^+$ T cells, stained with anti-CD8 (1.25 µg/mL in 5% rabbit serum; BioRad) as primary antibody and biotinylated rabbit anti-rat (1:200 in TBS; Vector Laboratories) as secondary antibody. After antibody staining, sections were incubated in ABC elite for 30 min at room temperature, developed with DAB ImmPACT kit (SK-4105, Vector Laboratories), and counterstained with Mayer's haematoxylin. Staining for IL1RAP in murine subvalvular plaques, cross-sections were stained with anti-IL1RAP (0.35 µg/mL in 2.5% goat serum, clone: 15F12) as primary antibody. After antibody staining, sections were incubated in ImmPRESS goat anti-rabbit (MP-7451, Vector) for 30 min at room temperature, developed with DAB ImmPACT kit (SK-4105, Vector Laboratories), and counterstained with Mayer's haematoxylin. For immunofluorescent staining of IL1RAP, CD68 macrophages and CD3 T cells, aortic root cross-sections were stained with anti-IL1RAP

(clone: 15F12) anti-CD3 (Armenian hamster anti-mouse, 100302, BioLegend) or anti-CD68 (clone FA-11) and fluorescently labeled (Alexa Fluor 488 & Alexa Fluor 555) secondary antibodies were added. After staining, sections were incubated in Sudan Black (0.03%) and counterstained with DAPI.
